# Definitions and outcomes reported in studies investigating the management of new onset atrial fibrillation in acutely unwell patients admitted to the intensive care unit: a scoping review

**DOI:** 10.1093/ehjopen/oeag075

**Published:** 2026-05-09

**Authors:** Brian W Johnston, Joby Mathew, Calum Voller, Ruaraidh A Hill, Bronagh Blackwood, Karen Williams, Angela Hall, Keith Wilson, Trudie H Lobban, Gregory Y H Lip, Ingeborg D Welters

**Affiliations:** Department of Cardiovascular and Metabolic Medicine, Institute of Life Course and Medical Sciences, University of Liverpool, 6 West Derby Street, Liverpool L78TX, UK; Liverpool Centre for Cardiovascular Science at University of Liverpool, Liverpool John Moores University, William Henry Duncan Building, 6 West Derby Street, Liverpool L7 8TX, UK; University Hospitals of Liverpool Group, Royal Liverpool University Hospital, Department of Critical Care Medicine, Mount Vernon Street, Liverpool L78YE, UK; Liverpool School of Medicine, University of Liverpool, Cedar House, Ashton Street, Liverpool L69 3GE, UK; Liverpool School of Medicine, University of Liverpool, Cedar House, Ashton Street, Liverpool L69 3GE, UK; Liverpool Reviews and Implementation Group (LRiG), University of Liverpool, Department of Health Data Science, Institute of Population Health, Waterhouse Building, Block B, Brownlow Building, Block B, Liverpool L69 3GF, UK; Wellcome Wolfson Institute for Experimental Medicine, Queens University Belfast, 97 Lisburn Road, Belfast BT9 7BL, UK; University Hospitals of Liverpool Group, Royal Liverpool University Hospital, Department of Critical Care Medicine, Mount Vernon Street, Liverpool L78YE, UK; University Hospitals of Liverpool Group, Royal Liverpool University Hospital, Department of Critical Care Medicine, Mount Vernon Street, Liverpool L78YE, UK; Liverpool Heart and Chest Hospital NHS Foundation Trust, Thomas Drive, Liverpool L14 3PE, UK; Arrhythmia Alliance, Heart Rhythm Alliances and STARS, Celixir House, Stratford Business and Technology Park, Innovation Way, Stratford upon Avon, Warickshire CV37 7GZ, UK; Department of Cardiovascular and Metabolic Medicine, Institute of Life Course and Medical Sciences, University of Liverpool, 6 West Derby Street, Liverpool L78TX, UK; Liverpool Centre for Cardiovascular Science at University of Liverpool, Liverpool John Moores University, William Henry Duncan Building, 6 West Derby Street, Liverpool L7 8TX, UK; Department of Clinical Medicine, Aalborg University, Fredrik Bajers Vej 7K, 9920 Aalborg, Denmark; Medical University of Bialystok, Jana Killnskiego 1, 15-089 Bialystok, Poland; Department of Cardiovascular and Metabolic Medicine, Institute of Life Course and Medical Sciences, University of Liverpool, 6 West Derby Street, Liverpool L78TX, UK; Liverpool Centre for Cardiovascular Science at University of Liverpool, Liverpool John Moores University, William Henry Duncan Building, 6 West Derby Street, Liverpool L7 8TX, UK; University Hospitals of Liverpool Group, Royal Liverpool University Hospital, Department of Critical Care Medicine, Mount Vernon Street, Liverpool L78YE, UK

**Keywords:** New-onset atrial fibrillation, Critical care, Scoping review, Outcome measures, Core outcome set, ISPOR

## Abstract

New-onset atrial fibrillation (NOAF) frequently occurs in critically ill patients admitted to intensive care units (ICUs), yet definitions and outcome reporting remain inconsistent across clinical studies. This heterogeneity impedes evidence synthesis and limits the development of standardized management strategies. A scoping review was conducted following the Arksey and O’Malley framework and reported using PRISMA-ScR guidelines. Electronic databases were searched from 1990 to 2024 for randomized controlled trials and observational studies reporting on NOAF in acutely unwell ICU patients. Data on arrhythmia definitions and reported outcomes were extracted and categorized using the ISPOR framework. This is the first study to systematically examine and compare diagnostic criteria for NOAF in ICU-based research. Out of 8477 records, 44 studies (4 RCTs, 12 prospective, and 28 retrospective observational studies) met inclusion criteria. Significant heterogeneity was found in how NOAF was defined, including arrhythmia duration thresholds, diagnostic criteria, and heart rate cut-offs. Outcomes were primarily clinician-reported (*n* = 101), followed by observer-reported (*n* = 74), biomarker (*n* = 25), and patient-reported outcomes (*n* = 4), with no performance outcomes identified. Common outcomes included rhythm and heart rate control, arrhythmia recurrence, mortality, and length of stay, though definitions and timeframes varied considerably across studies. This review identifies substantial methodological variation in both the definition and measurement of NOAF in ICU research. The findings are immediately applicable to clinical practice, enabling ICU teams to benchmark diagnostic and monitoring approaches, align practice with guideline-based definitions, and identify patients who may benefit from structured post-discharge follow-up. They also provide evidence for the importance of the development a Core Outcome Set for NOAF.

## Introduction and background

Atrial fibrillation (AF) is the most common cardiac arrhythmia and is estimated to affect approximately 33 million people worldwide.^1^ AF is defined by the European Society of Cardiology (ESC) as, ‘a supraventricular tachyarrhythmia with uncoordinated atrial electrical activation and consequently ineffective atrial contraction’.^1^ Electrocardiographic (ECG) features characteristic of the diagnosis of AF include irregular R–R intervals, absence of distinct repeating p-waves, and irregular atrial activation lasting at least 30 s.^1^ New onset atrial fibrillation (NOAF) is defined as AF developing in patients with no past medical history of AF.^1^

NOAF affects approximately 5–11% of all acutely unwell patients admitted to the intensive care unit (ICU).^2,3^ However, in subgroups including those admitted with septic shock, NOAF can affect up to 46% of patients.^3^ NOAF is not a benign entity, and its development is associated with short-term and long-term consequences such as haemodynamic instability, increased ICU and hospital mortality, increased ICU length of stay (LoS) and hospital LoS, development of permanent AF and cerebrovascular events.^3–7^

National and international guidelines have been published on the management of AF.^1,8–10^ However, they are not directly applicable to acutely unwell ICU patients and recent international surveys have highlighted differences between guidelines and clinicians’ management of NOAF in the ICU.^11,12^ Moreover, several systematic reviews, including over 50 clinical studies in acutely ill ICU patients, highlight significant variation in the definition of clinically relevant NOAF and heterogeneity in commonly reported outcome measures.^13–15^

The recent Symposium on Atrial Fibrillation in Acute and Critical Care highlighted the need for a consensus on a core outcome set for clinical trials, agreement on a definition of NOAF and further randomized controlled trials (RCTs) as key research priorities for AF in acutely unwell patients^16^ A core outcome set (COS) is an agreed set of outcomes that should be reported in all trials investigating areas of healthcare or specific healthcare conditions.^17,18^ Using COS aims to reduce inconsistency between trials by producing a minimum set of outcomes that should be included in clinical studies to allow comparisons between interventions.^17^

In this study, we systematically mapped outcomes reported in recent clinical trials investigating NOAF in acutely unwell adults admitted to the ICU. This work updates a recent systematic review^13^ with the aim of scoping the literature to provide a clear understanding of what definitions and outcomes are being reported in randomized and observational clinical trials investigating NOAF as a first step in the development of a COS to achieve consistency in reporting and reduction in heterogeneity. In addition, we mapped the variability in diagnostic approaches for NOAF used in clinical trials, including arrhythmia duration thresholds, heart rate criteria, and monitoring modalities. This work builds on our previous systematic review, which examined management strategies for NOAF in critically ill patients. In contrast, the present study focuses specifically on how NOAF is defined and how outcomes are reported, with the aim of informing the development of a core outcome set.

## Methods

### Design

This review follows the methodology introduced by Arksey and O’Malley for scoping reviews.^19^ We present our findings according to the five stages of the Arksey and O’Malley framework and describe identification of the research question, identification of relevant studies, study selection, study collation, summarizing and reporting of the results.^20^

We report the findings of this scoping review following the Preferred Reporting Items for Systematic reviews and Meta-Analyses extension for Scoping Reviews (PRIMSA-ScR) checklist.^21^

### Identifying the research question

The major aim of this scoping review is to rapidly map the literature to inform the future development of a COS. Our research priorities are to report the definitions of NOAF and the variation in arrhythmias included in clinical trials in critically unwell patients and to systematically map reported outcomes and outcome measures used in recent interventional and observational studies on NOAF in acutely unwell patients.

### Identifying relevant studies

The review focuses on NOAF in acutely unwell patients admitted to ICU. The search strategy of a previous systematic review published by our group was updated.^13,22^ We developed our search strategy with a specialist medical librarian (AH). Electronic databases, including Cochrane Central Register of Controlled Trials (CENTRAL), Embase (OVID), Emcare (OVID), and MEDLINE, were searched for studies published from 1990 to 2024. Reference lists of all included studies, systematic reviews, and meta-analyses, and the authors’ personal collections were also screened for relevant studies. We anticipated to include high-quality randomized controlled trials (RCTs); therefore, we included any RCT design, prospective observational studies, and retrospective observational studies.

Acutely unwell patients may present to several different specialities in healthcare settings including Emergency Medicine, Surgery or peri-operatively, Acute Medicine, and directly to Critical Care or ICU. Accordingly, we included all these speciality areas in our search strategy. The focus of our review was on patients admitted to the ICU, therefore only studies in which patients were admitted to the ICU were included in our final analysis. We did not limit our searches by language or publication status. The search strategy is presented in Supplementary material online, Table  *S1* (see Supplementary material). We excluded studies reporting specialist ICU populations such as neurosurgical and cardiac intensive care units.

### Study selection

Search results were exported to the Covidence systematic review platform (Veritas Health Innovation, Melbourne, Australia). Following duplicate removal, titles and abstracts were screened for eligibility against predetermined inclusion and exclusion criteria by two authors independently (JM and CV). Conflicts were resolved through discussion by a third senior review author (BWJ). Full-text articles were retrieved and screened against inclusion and exclusion criteria. Articles were then stratified based upon the predominant clinical setting, including emergency medicine, surgery, or perioperative, acute medicine, and critical care or ICU. Intensive Care Society Guidelines defining level 2 and level 3 care were used to identify studies whose population were predominantly admitted to ICU, which were progressed to data extraction. Disagreements were resolved through discussion and consensus with a senior reviewer (BWJ and IW).

### Data extraction and charting the data

Data were extracted using a custom data extraction form generated in Microsoft Excel (Microsoft Corp., Redmond, WA, USA). Extracted data included quantitative and qualitative data and included baseline study information (authors, year, region, funding), study design, study population characteristics, arrhythmias included in the study, and definition of arrhythmias were provided, interventions under investigation, primary and secondary outcomes, outcome measurement tools used. In addition to individual primary and secondary outcomes, we extracted the specific measurement variable, analysis metric, method of aggregation, and time point for each outcome as per SPIRIT 2013 definition.^23^

The International Society for Pharmacoeconomics and Outcomes Research (ISPOR) framework was used to categorize extracted outcomes.^24^ The ISPOR categorizes outcomes as either performance outcomes, observer-reported outcomes, clinician-reported outcomes, patient-reported outcomes, or biomarker outcomes. The definitions of the ISPOR framework categories are shown in Supplementary material online, *Table S2* (see Supplementary material).

We anticipated that studies would differ in the definition of outcomes used; we report each outcome definition and the frequency with which it is used.

### Collating, summarizing, and reporting the data

Individual study characteristics are reported in *Table 1*. We have systematically mapped the individual arrhythmias included in clinical trials, and when provided, the characteristics used to define AF and NOAF (*Table 2*). In addition, we report the method used for the diagnosis of NOAF (e.g. clinician, ECG) and any time frames or minimum arrhythmia duration as reported by the study authors (*Table 2*).

**Table 1:** Study characteristics and primary and secondary outcomes

	Author Year	Country	Mixed ICU	Medical ICU	Surgical ICU	Other	Study design	N Analysed	Arrhythmia	Funding	Primary outcomes	Secondary Outcomes
**Randomized controlled trials**												
**1**	**Kakihana *et al.* (2020)** ^25^	Japan	•				Multicentre open-label randomized controlled trial	151	Atrial fibrillation Atrial flutter SVT	Ono Pharmaceutical Co.	HR control (defined as HR 60–94bpm after 24 h)	Decrease in HR >20% or more at 24 h, 48 h, 72 h and 96hr ICU free days at 28d Hospital free days at 28d 28d mortality Lactate, creatinine kinase, troponin-I, BNP, arterial pH, BE, PF ratio, PaCO2, eGFR
**2**	**Delle Karth *et al.* (2001)** ^26^	Austria		•			Randomized controlled trial	60	Atrial fibrillation Atrial flutter Atrial tachycardia	*nn/r*	Cardioversion to SR	HR reduction >30% of baseline for within a 4 h period and sustained for >4 h Controlled tachycardia (HR <120) Uncontrolled tachycardia (HR >120 within 4 h of study drug administration)
**3**	**Balser *et al.* (1998)** ^27^	USA			•		Randomized controlled trial	64	Atrial fibrillation Atrial flutter Unspecified SVT	*nn/r*	Cardioversion to SR at 2 h and 12 h	Heart rate (reported but no definition of successful heart rate control) ICU LoS Hospital mortality
**4**	**Moran *et al.* (1995)** ^28^	Australia		•			Randomized controlled trial	42	Atrial tachyarrhythmia (unspecified) for >1 h	Queen Elizabeth Hospital ICU, Woodville departmental funds	Cardioversion to SR within 24 h Heart rate (reported but no definition of successful heart rate control)	*nn/r*
**Prospective observational studies**												
**5**	**Uchino *et al.* (2020)**	Japan	•				Multicentre prospective observational cohort study	412	NOAF	The Jikei University Research Fund	In hospital mortality in NOAF lasting >48 h vs. NOAF lasting <48 h	Hospital LoS Incidence of stroke
**6**	**Meierhenrich *et al.* (2010)** ^29^	Germany			•		Single centre prospective observational	49	NOAF in patients with and without septic shock patients	*nn/r*	Cardioversion to SR	Arrhythmia recurrence rate ICU mortality 28d and 60d CRP, WCC
**7**	**Gerlach *et al.* (2008)** ^30^	USA			•		Single-centre prospective observational cohort study	61	Atrial tachycardia Atrial fibrillation Atrial flutter	*nn/r*	Cardioversion to SR at 24 h	Time to cardioversion
**8**	**Milicevic *et al.* (2008)** ^31^	Croatia		•			Single-centre prospective observational cohort study	140	NOAF (<72 h)	Ministry of Science, Education and Sport, of the Republic of Croatia	Cardioversion to SR following sequential administration of up to 3 antiarrhythmics	Time to cardioversion to SR
**9**	**Sleeswijk *et al.* (2008)** ^32^	Netherlands	•				Single-centre prospective observational	29	Atrial fibrillation	*nn/r*	Cardioversion to SR	HR control (<110 bpm) Time to cardioversion or HR control
**10**	**Hayashi *et al.* (2005)** ^33^	Japan	•				Single-centre prospective observational	84	Haemodynamically unstable NOAF	*nn/r*	Cardioversion to SR following DCCV failure (failure to maintain cardioversion following 3xDCCV shocks)	*nn/r*
**11**	**Mayr *et al.* (2003)** ^34^	Austria			•		Single centre	37	Atrial fibrillation SVT	*nn/r*	Cardioversion to SR	*nn/r*
**12**	**Hennersdorf *et al.* (2002)** ^35^	Germany	•				Single-centre prospective observational	26	Atrial fibrillation Atrial flutter	*nn/r*	Termination of arrhythmia in <90 min of Ibutilide infusion	*nn/r*
**13**	**Song *et al.* (2023)** ^36^	Seoul^b^	•				Propensity-matched cohort study	22237	NOAF	Seoul National University Bundang Hospital	Occurrence of NOAF with 7d of ICU admission	Time from ICU admission to NOAF ICU length of stay Hospital length of stay In hospital mortality Adverse events associated with Dexmedetomidine treatment of AF
**14**	**Makrygiannis *et al.* (2018)** ^37^	Greece	•				Single-centre retrospective study	133	NOAF	*nn/r*	All-cause mortality during ICU	ICU length of stay
**15**	**Arrigo *et al.* (2018)** ^38^	France Belgium	•				Multicentre prospective cohort study	1841	NOAF PEAF No AF	No funding declared	All-cause 1 year mortality	Incidence of cardiovascular morbidity Incidence of cardiovascular mortality Quality of life 1 year post ICU^c^ Biomarkers in long term outcome post ICU^d^
**16**	**Wetterslev *et al.* (2023)** ^7^	Denmark Netherlands Norway Finland Poland Sweden Switzerland Kingdom of Saudi Arabia China India Australia New Zealand	•				Multicentre prospective, inception cohort study	1423	NOAF	The Research Council of Rigshospitalet. The Danish Society of Anaesthesiology and Intensive Care Medicine. The Ehrenreich’s foundation.	Frequency of AF and NOAF	Length of hospital stay Ischaemia or thromboembolic events Severe bleeding event 90-day mortality
**Retrospective observational studies**												
**17**	**Rottmann *et al.* (2024)** ^39^	Germany		•			Single centre retrospective cohort	616	NOAF PEAF	Deutsche Forschungsgemeinschaft (DFG, German Research Foundation)	ICU survival	Hospital survival Length of stay Readmission to ICU Hours of mechanical ventilation Use of ECMO Needs for dialysis
**18**	**Bedford *et al.* (2022)** ^40^	UK	•				Multi-centre retrospective cohort study^e^	1200	NOAF	National Institute for Health Research. Health Technology Assessment programme	Time to rate control (HR <110 bpm) Time to rhythm control	0-day mortality
**19**	**Gillmann *et al.* (2022)** ^41^	Germany	•				Single-centre retrospective cohort study	8611	NOAF	Projekt DEAL	Time to reach HR <110 bpm for 1 h	Heart rate delta relative to medication start Hours in sinus rhythm within first 24 h Hours until sinus rhythm conversion Vital parameters at sinus rhythm conversion Changes in haemodynamic stability Occurrence of bradycardic episodes
**20**	**Miller *et al.* (2022)** ^42^	UK	•				Single-centre retrospective cohort study	97	NOAF PEAF	No funding declared	Incidence of therapeutic anticoagulation	Thromboembolic events Bleeding events ICU length of stay Hospital length of stay Basic/advanced respiratory support duration Basic/advanced cardiac support duration ICU mortality Hospital mortality HASBLED score CHADSVASc score
**21**	**Suresh *et al..* (2022)** ^43^	USA		•			Single-centre retrospective cohort study	2491	NOAF	*nn/r*	Conversion to sinus rhythm	AF recurrence in 24 h AF recurrence after 24 h of SR Adverse events: CVA Encephalopathy Myocardial infarction 30-day mortality Drug related adverse events
**22**	**Shima *et al.* (2021)** ^44^	Japan	•				Sub-analysis of AFTER-ICU study	65	NOAF	Jikei University Research Fund	Restoration of sinus rhythm following DCCV	ICU mortality Hospital mortality Cardiac rhythm at ICU discharge ICU length of stay Hospital length of stay Ischaemic stroke Bleeding events
**23**	**Yoshida *et al.* (2021)** ^45^	Japan	•				Sub-analysis of AFTER-ICU study	423	NOAF	Jikei University Research Fund	Last SR restoration (within 7 days after the initial AF onset)	Cardiac rhythm at ICU discharge AF duration ICU length of stay Hospital length of stay ICU mortality Hospital mortality In hospital stroke
**24**	**Jacobs *et al.* (2020)** ^46^	Netherlands	•				Single-centre Retrospective cohort	3334	NOAF PEAF	No external funding declared	In-hospital mortality	1 year mortality Morality at maximum follow up ICU length of stay Hospital length of stay
**25**	**Betthauser *et al.* (2019)**	USA	•				Single-centre retrospective observational cohort study	239	Atrial fibrillation	None declared	Descriptive use of amiodarone	ICU LoS
**26**	**Herasevich *et al.* (2019)** ^47^	USA	•				Single-centre retrospective observational cohort study	180	Tachycardia associated with sepsis including: Atrial fibrillation Atrial flutter Sinus tachycardia SVT Atrial tachycardia	Integrated Multidisciplinary Practice, Mayo Clinic, Rochester	Impact of digoxin on haemodynamic profile (including HR pre and post digoxin at 6, 12, 24 h)	Hospital mortality Hospital LoS ICU mortality ICU LoS
**27**	**Kim *et al.* (2019)** ^48^	South Korea	•				Retrospective cohort study^a^	30869	NOAF	National Research Foundation of Korea	First occurrence of ischaemic stroke/systemic emboli	All-cause mortality 6-month mortality
**28**	**Kyo *et al.* (2019)** ^49^	Japan	•				Two-centre retrospective cohort	85	NOAF	No external funding declared	ICU mortality	DCCV energy Number of shocks during DCCV Time from AF to DCCV Success rate of DCCV Recurrence of AF after DCCV Cerebral infarction rate Length of hospital stay Length of ICU stay
**29**	**Milojevic *et al.* (2019)** ^50^	France				•	Three mobile intensive care unit	300	Atrial fibrillation	*nn/r*	Cardioversion to SR within 40 min of treatment initiation	HR <100 within 40 min of treatment initiation
**30**	**Schoaps *et al.* (2019)** ^51^	USA	•				Single-centre retrospective cohort study	640	NOAF	*nn/r*	Prescription of oral anticoagulant on hospital discharge (in NOAF with CHA_2_DS_2_VASc >2)	ICU length of stay Hospital length of stay Reason for non-prescription OAC Association between individual CHA_2_DS_2_VASc components and OAC prescription
**31**	**Brown *et al.* (2018)** ^52^	USA			•		Single-centre Retrospective observational	33	NOAF with HR >100 bpm	National Institutes of Health and the NHLBI award: 1T35HL120835	Cardioversion to SR within 48	HR control (reported but no definition of successful heart rate control) IU LoS Hospital LoS
**32**	**Yoshida *et al.* (2018)** ^53^	Japan	•				Single-centre retrospective observational cohort study	151	NOAF	*nn/r*	Cardioversion to SR within 6 h	In-hospital mortality Incidence of stroke
**33**	**Balik *et al.* (2017)** ^54^	Prague	•				Single-centre retrospective observational	234	Supraventricular arrhythmia	OP Prague Competitiveness	Cardioversion to SR within 24 h	ICU mortality 28d mortality 12-month mortality
**34**	**Duby *et al.* (2017)** ^55^	USA	•				Single-centre retrospective observational	191	Atrial fibrillation	National centre for Advancing Translational Sciences, National Institutes of Health: UL1 TR00002	Mortality	ICU LoS Hospital LoS Thromboembolic events (PE/DVT/Stroke)
**35**	**Liu *et al.* (2016)** ^56^	Taiwan		•			Single-centre retrospective observational	503	NOAF in sepsis	Ministry of Defence, Republic of China (MAB-105–014)	Cardioversion to SR	In hospital mortality
**36**	**Mitric *et al.* (2016)** ^57^	Netherlands	•				Single-centre retrospective observational	186	Atrial fibrillation	*nn/r*	Cardioversion to SR within 12 h	*nn/r*
**37**	**Walkey *et al.* (2016)** ^58^	USA	•				Multicentre retrospective propensity-matched cohort study	39693	Atrial fibrillation developing during sepsis	None declared	In hospital mortality	*nn/r*
**38**	**Okajima *et al.* (2015)** ^59^	Japan	•				Single-centre retrospective observational	61	SVT (HR >120): Paroxysmal AF Paroxysmal atrial flutter Paroxysmal atrial tachycardia Paroxysmal supraventricular tachyarrhythmia	*nn/r*	Cardioversion to SR	HR reduction without decreases in arterial pressure (successful HR reduction not defined)
**39**	**Xie *et al.* (2015)** ^60^	China	•				Single-centre retrospective observational	2586	NOAF	*nn/r*	Cardioversion to SR	30-day mortality
**40**	**Personett *et al.* (2014)** ^61^	USA	•				Single-centre retrospective observational cohort study	121	Atrial fibrillation Atrial flutter	Department of Pharmacy Services, Department of Trauma, Critical Care and General Surgery, Mayo Clinic, Rochester.	HR control (defined as HR <110 beats/min and SBO >90 mmHg for 6hrs	ICU LoS Readmission to ICU for AF
**41**	**Kanji *et al.* (2012)** ^62^	Canada	•				Multicentre retrospective observational	348	Atrial fibrillation Atrial flutter	*nn/r*	Rhythm control/HR control	In hospital mortality ICU mortality Hospital LoS ICU LoS Thromboembolic/Stroke
**42**	**Delle Karth (2005)** ^63^	Vienna, Austria		•			Single-centre retrospective observational	17	Atrial fibrillation Atrial flutter	*nn/r*	Termination of arrhythmia within 60 min	*nn/r*
**43**	**Mayr *et al.* (2004)** ^64^	Austria	•				Single-centre retrospective observational	70	New onset SVT	*nn/r*	Haemodynamics and cardioversion to SR Heart rate at 12, 24, and 48 h	*nn/r*
**44**	**Varriale *et al.* (2000)** ^65^	USA	•				Single-centre retrospective observational	34	Atrial fibrillation Atrial flutter	*nn/r*	Cardioversion to SR	*nn/r*

Abbreviations: SR sinus rhythm, SVT supraventricular tachycardia, HR heart rate, ICU intensive care unit, LoS length of stay, NOAF new onset atrial fibrillation, PEAF pre-existing atrial fibrillation, PE pulmonary embolism, DVT deep vein thrombosis, DCCV direct current cardioversion, CCB calcium channel antagonist, *nn/r* not reported.

^a^Based on two retrospective cohorts: (i) AF-cohort from National Health Insurance Service of South Korea and (ii) validation cohort from two centre hospital cohorts with AF.

^b^Study team based in Seoul but utilized the Medical Information Mart for Intensive Care (MIMIC)-IV database for the primary analysis.

^c^Hospital Anxiety and Depression Scale (HADS), Short Form-36 (SF-36), Impact of Event Scale-Revised (IES-R) at 3, 6 and 12 months, social questionnaire assessing social environment at 3 months.

^d^Copeptine, Proenkephalin, Troponin I us, Troponin T hs, Brain naturetic peptide, N-terminal pro-BNP, Adrenomedullin, Soluble ST2, Galectine 3, CRP, Interleukin 6, Procalcitonin, Plasma cystatin C, Urinary cystatin C, plasma and urine neutrophil gelatinase-associated lipocalin.

^e^Analysis comprised cohorts from the Medical Information Mart for Intensive Care (MIMIC)-III database and Post-intensive Care Risk-adjusted and Monitoring Database (PICRAM).

**Table 2:** Definitions of arrhythmia, arrhythmia duration, heart rate criteria and measurement tool reported in studies

	Author Year	Type of arrhythmia included in analysis	Definition of included arrhythmias	Arrhythmia duration prior to intervention	Heart rate criteria for intervention	Measurement tool
**Randomised controlled trials**						
1	**Kakihana *et al.* (2020)**	Atrial fibrillation Atrial flutter SVT	*n*/*r*	*n*/*r*	>100bpm	12-lead ECG
2	**Delle Karth *et al.* (2001)** ^63^	Atrial fibrillation Atrial flutter Atrial tachycardia	Recent onset atrial fibrillation (no further definition)	>30 min	>120 bpm	12- lead ECG
3	**Balser *et al.* (1998)** ^27^	Atrial fibrillation Atrial flutter Unspecified SVT	*n*/r	15 min	>100bpm	12-lead ECG Rhythm strip prior to intervention
4	**Moran *et al.* (1995)** ^28^	Atrial tachyarrhythmia (unspecified)	*n*/r	>60min	>120bpm (ventricular response)	Continuous 3-lead ECG
**Prospective observational studies**						
5	**Uchino *et al.* (2020)**	NOAF	Irregular R-R intervals without apparent p-waves or with F waves	>5 min or with recurrent episode within 5 min	*n*/r	12 lead ECG or continuous 3 lead ECG
6	**Meierhenrich *et al.* (2010)** ^29^	NOAF in patients with and without septic shock patients	Loss of regular RR-interval Irregular ventricular activity Chaotic atrial activity No apparent p-wave	*n*/r	>110bpm	Continuous 3- lead ECG 12 lead ECG confirmation
7	**Gerlach *et al.* (2008)** ^30^	Atrial tachycardia Atrial fibrillation Atrial flutter	*n*/*r*	*n*/*r*	*nn/r*	*nn/r*
8	**Milicevic *et al.* (2008)** ^31^	NOAF (<72 h)	*nn/r*	*nn/r*	*nn/r*	12- lead ECG every 12 h
9	**Sleeswijk *et al.* (2008)** ^32^	Atrial fibrillation	Irregular, chaotic atrial activity without apparent p-waves and irregular ventricular activity	>30 min	>110bpm	12- lead ECG
10	**Hayashi *et al.* (2005)** ^33^	Haemodynamically unstable NOAF	*nn/r*	30 min	Haemodynamically unstable (not defined)	Connected to defibrillator but no report of how NOAF measured
11	**Mayr *et al.* (2003)**	Atrial fibrillation SVT	Narrow complex non sinus tachycardia	>15 min	>100 bpm	12-lead ECG
12	**Hennersdorf *et al.* (2002)** ^35^	Atrial fibrillation Atrial flutter	*nn/r*	*nn/r*	*nn/r*	*nn/r*
13	**Song *et al.* (2023)** ^36^	NOAF	MIMC-IV	MIMIC-IV	MIMIC-IV	MIMIC-IV
14	**Makrygiannis (2018)** ^37^	NOAF	R-R interval irregularity Absences of distinct p waves Replacement of p waves with fibrillatory waves	*nn/r*	*nn/r*	Continuous 3- lead ECG 12-lead ECG Diagnosis confirmed offline by cardiologist
15	**Arrigo *et al.* (2018)** ^38^	NOAF PEAF	NOAF: not previously documented PEAF: previous diagnosis	*nn/r*	*nn/r*	12—lead ECG
16	**Wetterslev *et al.* (2023)** ^7^	NOAF	Irregular rhythm with the absence of p waves, irregular RR intervals	>30s	*nn/r*	Continuous monitoring or 12-lead electrocardiogram (ECG) Confirmed by physician
**Retrospective observational studies**						
17	**Rottmann *et al.* (2024)** ^39^	NOAF PEAF	*nn/r*	*nn/r*	*nn/r*	12 -lead ECG
18	**Bedford *et al.* (2022)** ^40^	NOAF (PICRAM and MIMIC-III database)	*nn/r*	>30s	*nn/r*	Continuous 3-lead ECG Nursing notes
19	**Gillmann *et al.* (2022)** ^41^	NOAF	NOAF: as diagnosed by treating clinician	*nn/r*	>110bpm	*nn/r*
20	**Miller *et al.* (2022)** ^42^	NOAF PEAF	*nn/r*	*nn/r*	*nn/r*	ECG (unclear if 3-lead, continuous or 12-lead)
21	**Suresh *et al.* (2022)** ^43^	NOAF	Irregularly irregular rhythm Absence of p waves Variable ventricular rate	>30min	*nn/r*	Continuous telemetry (no further definition/lead number provided)
22	**Shima *et al.* (2021)** ^44^ **(AFTER-ICU reanalysis)**	NOAF	Irregular R-R intervals Without apparent p-waves (or) presence of F waves	>5 min	*nn/r*	12-lead ECG 3-lead ECG
23	**Yoshida *et al.* (2021)** ^45^	Sub-analysis of AFTER-ICU study	Irregular R-R intervals Without apparent p-waves (or) presence of F waves	>5 min	*nn/r*	12-lead ECG 3-lead ECG
24	**Jacobs *et al.* (2020)** ^46^	NOAF PEAF	ECG automatic analysis confirmed by cardiologist NOAF: no PMHs	*nn/r*	*nn/r*	12- lead ECG (automatic analysis)
25	**Betthauser *et al.* (2019)**	Atrial fibrillation	NOAF defined as AF in patient without prior cardiac arrhythmia	*nn/r*	*nn/r*	*nn/r*
26	**Herasevich *et al.* (2019)** ^47^	Tachycardia associated with sepsis including: Atrial fibrillation Atrial flutter Sinus tachycardia SVT Atrial tachycardia	*nn/r*	*nn/r*	>100 bpm	*nn/r*
27	**Kim *et al.* (2019)** ^48^	NOAF (ICU acquired AF)	ICU acquired AF: first reported AF (no further definition)	*nn/r*	*nn/r*	*nn/r*
28	**Kyo *et al.* (2019)** ^49^	NOAF	Clinician diagnosed	*nn/r*	*nn/r*	Bedside monitor 12- lead ECG Clinician diagnosed
29	**Milojevic *et al.* (2019)** ^50^	Atrial fibrillation	*nn/r*	*nn/r*	*nn/r*	*nn/r*
30	**Schoaps (2019)** ^51^	NOAF	*nn/r*	*nn/r*	*nn/r*	12- lead ECG
31	**Brown *et al.* (2018)** ^52^	NOAF	*nn/r*	*nn/r*	>100bpm	12-lead ECG interpreted by cardiologist
32	**Yoshida *et al.* (2018)** ^53^	NOAF	NOAF defined as AF in patient without history of AF	*nn/r*	*nn/r*	Continuous ICU ECG monitoring
33	**Balik *et al.* (2017)** ^54^	Supraventricular arrhythmia	*nn/r*	*nn/r*	*nn/r*	*nn/r*
34	**Duby *et al.* (2017)** ^55^	Atrial fibrillation	*nn/r*	*nn/r*	*nn/r*	*nn/r*
35	**Liu *et al.* (2016)** ^56^	NOAF in sepsis	Absence of p-waves and irregular ventricular activity	>30s	*nn/r*	12- lead ECG
36	**Mitric *et al.* (2016)** ^57^	Atrial fibrillation	Replacement of p-waves with rapid oscillatory or fibrillatory waves that vary in shape, size and timing, associated with irregular, frequent rapid ventricular response when AV conduction is intact	1 h	*nn/r*	Continuous ECG (Philips Intelli Vue IP monitor)
37	**Walkey *et al.* (2016)** ^58^	Atrial fibrillation developing during sepsis	*nn/r*	*nn/r*	*nn/r*	*nn/r*
38	**Okajima *et al.* (2015)** ^59^	SVT defined as: Paroxysmal AF Paroxysmal atrial flutter Paroxysmal atrial tachycardia Paroxysmal supraventricular tachyarrhythmia	*nn/r*	> 1 h	>120bpm	*nn/r*
39	**Xie *et al.* (2015)** ^60^	NOAF				
40	**Personett *et al.* (2014)** ^61^	Atrial fibrillation Atrial flutter	*nn/r*	*nn/r*	>110bpm*(retrospective analysis but treatment success <110bpm).	*nn/r*
41	**Kanji *et al.* (2012)** ^62^	Atrial fibrillation Atrial flutter	NOAF defined as AF without PMH of AF	*nn/r*	>100bpm	*nn/r*
42	**Delle Karth (2005)**	Atrial fibrillation Atrial flutter	*nn/r*	Recent onset >1 h Sustained >10 min	>100 bpm	Continuous rhythm strip 12 lead ECG at baseline
43	**Mayr *et al.* (2004)** ^64^	New onset SVT	Narrow complex, non-sinus tachycardia	>30min	>100bpm	12-lead ECG
44	**Varriale *et al* (2000)** ^65^	Atrial fibrillation Atrial flutter	*nn/r*	*nn/r*	*nn/r*	12-lead ECG

Primary and secondary outcomes extracted from included studies are presented in Supplementary material online, *Table S3* (see Supplementary material). Outcomes were classified using the framework by the International Society for Pharmacoeconomics and Outcomes Research (ISPOR).^24^ Individual primary and secondary outcomes were grouped according to the domains proposed in the ISPOR framework^24^ and presented in *Table 3*. In addition, we report the outcome assessment as the measuring instrument providing a rating or score that represents the concept of interest ‘outcome’ of measurement.^24^ Clinical outcome assessments include observer-reported outcomes, performance outcomes, patient-reported outcomes, and clinician-reported outcomes.^24^ Quantitative outcome data will be analysed narratively. Individual outcomes will be described as counts and the frequency with which they are used in clinical trials will be grouped by ISPOR category. Outcome counts reflect the number of individual outcome assessments reported across studies, rather than unique outcome domains.

**Table 3:** ISPOR outcome assessments reported across 44 trials on the management of atrial fibrillation in acutely unwell adults

Type of outcome assessment	Total times reported across all trials	Outcome assessments included as primary or secondary study outcomes (*n* = number of trials reporting individual outcome assessment)
**Observer-reported outcome (ObsRO)**	74	**Mortality:** Morality (not defined) (*n* = 1) ICU mortality (*n* = 9) In-hospital mortality (*n* = 13) In-hospital morality (stratified by NOAF lasting <48 h and NOAF lasting >48 h) (*n* = 1) 28d mortality (*n* = 3) 30d mortality (*n* = 3) 60d mortality (*n* = 1) 90d day mortality (*n* = 1) All-cause mortality during ICU (*n* = 1) 6-month mortality (*n* = 1) 1-year mortality (*n* = 2) Mortality at maximum follow-up (*n* = 1) All-cause mortality (*n* = 1) All-cause mortality at 1-year (*n* = 1) **Survival:** Hospital survival (*n* = 1) ICU survival (*n* = 1) **Length of stay** ICU length of stay (*n* = 16) Hospital length of stay (*n* = 14) ICU free days at 28 days (*n* = 1) Hospital free days at 28 days (*n* = 1) Readmission to ICU (*n* = 1)
**Clinician-reported outcome (ClinRO)**	101	**Heart rate control or decrease:** HR control (60–94bpm) within 24 h (*n* = 1) Heart rate control (not defined by study authors) (*n* = 2) Time to heart rate control (heart rate control not defined by study authors) (*n* = 1) Time to HR control (<110bpm) (*n* = 1) Decrease in heart rate > 20% (24 h, 48 h, 72 h, 96 h) (*n* = 1) HR reduction >30% for 4 h within 4 h (*n* = 1) Controlled tachycardia (<120bpm) (*n* = 1) Uncontrolled tachycardia (>120bpm) (*n* = 1) HR control (<110bpm) and SBP >90 mmHg for 6 h (*n* = 1) HR control (<100bpm) (*n* = 1) HR control (<110bpm) (*n* = 1) HR at 12, 24 and 48 h (*n* = 1) HR reduction without decrease in arterial pressure (successful HR reduction not defined) (*n* = 2) **Cardioversion or rhythm control:** Cardioversion to sinus rhythm (*n* = 9) Cardioversion to sinus rhythm within 40 min (*n* = 1) Cardioversion to sinus rhythm at 2 h and 12 h (*n* = 1) Cardioversion to sinus rhythm at 6 h (*n* = 1) Cardioversion to sinus rhythm at 12 h (*n* = 1) Cardioversion within 12 h (*n* = 1) Cardioversion to sinus rhythm at 24 h (*n* = 3) Cardioversion to sinus rhythm in 48 h (*n* = 1) Cardioversion following DCCV failure (*n* = 1) Time-to-cardioversion to sinus rhythm (*n* = 1) Time to rhythm control (*n* = 1) Success rate of DCCV (success not defined) (*n* = 3) Rhythm control (*n* = 1) **Arrhythmia Recurrence:** Arrhythmia recurrence rate (*n* = 1) Arrhythmia recurrence after 24 h sinus rhythm (*n* = 1) AF recurrence in 24 h (*n* = 1) AF recurrence after DCCV (*n* = 1) **Stroke and anticoagulation:** Incidence of stroke (*n* = 7) First occurrence of ischaemic stroke or systemic emboli (*n* = 1) Thromboembolic events (*n* = 4) Bleeding events (*n* = 3) HASBLED score (*n* = 1) CHA_2_DS_2_VASc score (*n* = 1) Prescription of oral anticoagulant (*n* = 1) Prescription of oral anticoagulant on hospital discharge (*n* = 1) Incidence of therapeutic anticoagulation (*n* = 1) **Other misc. ClinRO reported specific to individual trial procedures:** Termination of arrhythmia within 60 min of antiarrhythmic medication (*n* = 1) Termination of arrhythmia following ibutilide infusion (*n* = 1) Impact of digoxin on haemodynamics (HR and BP) at 6, 12 and 24 h) (*n* = 1) Incidence of cardiovascular morbidity (not defined by study authors) (*n* = 1) Incidence of cardiovascular mortality (not defined by study authors (*n* = 1) Hours of mechanical ventilation (*n* = 1), need for dialysis (*n* = 1), need for ECMO (*n* = 1) DCCV energy (*n* = 1), number of shocks during DCCV (*n* = 1), time from AF-to-DCCV (*n* = 1) Time from ICU admission to NOAF (*n* = 1) Heart rate delta relative to medication start (*n* = 1) Descriptive use of amiodarone (*n* = 1) Readmission due to ICU for AF (*n* = 1) Haemodynamically unstable NOAF (*n* = 1) Changes to haemodynamic stability (*n* = 1) Cardiac rhythm at ICU discharge (*n* = 2) AF duration (*n* = 1), AF treatment (*n* = 1) Post antiarrhythmic: Change in heart rate following antiarrhythmic (*n* = 1), hours in sinus over 24 h (*n* = 1), hours until sinus cardioversion (*n* = 1), vital parameters at sinus rhythm conversion (*n* = 1), changes in haemodynamic stability (*n* = 1), Occurrence of bradycardic episodes (*n* = 1), incidence of therapeutic anticoagulation (*n* = 1), basic/advanced respiratory support requirements (*n* = 1), descriptive treatment of AF (*n* = 1), reason for non-prescription of anticoagulation (*n* = 1), association of CHA_2_DS_2_VASc and anticoagulation prescription (*n* = 1) Occurrence of NOAF in first 7d ICU admission (*n* = 1), Time from ICU admission to NOAF (*n* = 1), Adverse effects of dexmedetomindine (*n* = 1), drug related adverse events (*n* = 1) Myocardial infarction (*n* = 1), cerebrovascular accident (*n* = 1), Encephalopathy (*n* = 1) Cardioversion to SR following sequential administration of up to 3 antiarrhythmics (*n* = 1) Last SR restoration (within 7 days after the initial AF onset) (*n* = 1)
**Patient-reported outcome (PRO)**	4	Hospital Anxiety and Depression Scale (HADS) (*n* = 1) Short Form-36 (SF-36) (*n* = 1) Impact of Event Scale-Revised (IES-R) at 3, 6 and 12 months (*n* = 1) Social questionnaire assessing social environment at 3 months (*n* = 1)
**Performance-reported outcome**	0	
**Biomarker outcome**	25	Lactate (*n* = 1), Creatinine kinase (*n* = 1), Troponin I (*n* = 1), BNP (*n* = 1), arterial pH (*n* = 1), base excess (*n* = 1), PF ratio (*n* = 1), PaCO_2_ (*n* = 1), eGFR (*n* = 1), White cell count (*n* = 1) Co-peptine (*n* = 1), Proenkephalin (*n* = 1), Troponin T (*n* = 1), N-terminal-pro-BNP (*n* = 1), Adrenomedullin (*n* = 1), Soluble ST2 (*n* = 1), Galectine 3 (*n* = 1), Interleukin 6 (*n* = 1), Procalcitonin (*n* = 1), Plasma cystatin C (*n* = 1), Urinary cystatin C (*n* = 1), Plasma neutrophil gelatinase (*n* = 1), Urine neutrophil gelatinase (*n* = 1), Liopcalin (*n* = 1) CRP (*n* = 2)

## Results

Our search identified 8477 individual citations. Following duplicate removal, 6505 citations were screened by title and abstract, with 245 citations progressing to full text screening. Following full text screening, 93 articles were stratified based upon the setting and patient population into the critical care or ICU setting (*n* = 44), the emergency setting (*n* = 34), the surgical and perioperative setting (*n* = 11), and the acute medical setting (*n* = 2).

Our primary focus was to identify articles whose population represented acutely unwell patients admitted to the ICU. Therefore, only those citations reporting a patient population in the critical care or ICU setting underwent full data extraction Supplementary material online, *Figure S1* (see Supplementary material).

### Study characteristics

Data were extracted from four randomized controlled trials (RCT),^25–28^ 12 prospective observational studies^7,29–36,66^ and 28 retrospective observational studies^37–65,67^ enrolling 118,744 individual patients. The study characteristics are presented in *Table 1*. The majority of included studies were conducted in Europe^7,26,28,29,31,32,34,35,37–42,46,50,54,57,63,64^(*n* = 19), followed by the USA^27,30,43,47,51,52,55,58,61,65,67^ (*n* = 11) and Japan^25,33,44,45,49,53,59,66^ (*n* = 8). Two trials were conducted in South Korea^36,48^ while two trials were conducted in China,^7,60^ Australia,^7,28^ and one in Taiwan,^56^ Canada,^62^ Saudia Arabia,^7^ and India,^7^ respectively. The majority of studies (*n* = 30) were conducted in ICUs receiving mixed medical and surgical patients,^7,25,32–38,40–42,44–46,48,49,51,53–55,57–62,64,65,68,69^ followed by ICUs receiving predominantly separate medical^26,28,31,32,56,63^ (*n* = 5) and surgical^27,29,30,34,52^ (*n* = 5) cohorts. Two studies used data from the MIMIC database^36,40^ and the PICRAM database,^40^ while one study described including patients from a mobile ICU in France^50^ and one from a mixed/specialist burns ICU.^43^

### Defining new-onset atrial fibrillation in clinical trials

A range of arrhythmias were reported by studies included in our review. New onset atrial fibrillation accounted for the majority of arrhythmias, followed by atrial fibrillation, atrial flutter, supraventricular tachycardia, atrial tachycardia, preexisting atrial fibrillation, and sinus tachycardia. Of the 44 trials, 19 provided a definition for the arrhythmias included in the study (*Table 2*). Few studies explicitly reported diagnostic criteria aligned with ESC guideline definitions of atrial fibrillation.^1^ Studies that included the term new onset atrial fibrillation defined this as AF in patients without a previous history of AF and closely aligns with the ESC terminology of first detected AF.^1^ One study used the term ICU-acquired AF to denote the first reported AF occurring in the ICU but provided no further information on whether episodes of AF outside the ICU were included. Three trials confirmed that the diagnosis was made by a cardiologist or clinician without further information as to how the diagnosis was made or which criteria were used.

No trials reported the duration of AF required for the diagnosis of AF. There was wide variation in the duration of AF required prior to intervention in randomized or prospective trials. Delle Karth et al and Balser et al were the only RCTs to stipulate a duration of sustained AF required prior to randomization or intervention.^26,27^ Delle Karth et al required sustained AF of longer than 30 min prior to randomization, whilst Balser et al required 15 min of AF.^26^ Fourteen observational trials included a requirement of arrhythmia duration prior to intervention. The majority of trials reported 30 min of sustained AF as the cut-off prior to diagnosis or intervention; however, three studies reported a 1 h cut-off, while two studies used a 30 s cut-off for the diagnosis of AF.

A heart rate criterion was stipulated in fifteen studies prior to clinical intervention. A heart rate of 100 beats per minute was required in 8 studies, whilst a heart rate over 110 and 120 beats per minute was reported by 4 and 3 studies, respectively. The majority of studies did not report or require any heart rate criterion prior to diagnosis or intervention.

The use of ECG for NOAF diagnosis was extracted (*Table 2*). The majority (*n* = 29) reported using ECG for the diagnosis of AF. ECG recordings were predominantly standard 12-lead ECGs, but a number of studies used continuous 3-lead or continuous single-lead waveforms in addition to 12-lead ECGs for screening for AF. No studies reported patient symptoms or use of wireless monitoring, such as smart devices or continuous electronic implantable devices (CEID), as a screening tool.

### Reported outcomes according to ISPOR framework

Across 44 studies we identified, 101 clinician-reported outcome (ClinRO) assessments, 74 observer reported clinical outcome (ObsRO) assessments, 4 patient reported outcome (PRO) assessments, and 25 individual biomarker assessments. No studies included performance-reported outcome assessments (*Table 3*).

### Observer-reported outcomes

Observer-reported clinical outcome (ObsRO) measures were reported 74 times in the included studies. The most commonly reported ObsRO measurement categories included mortality, survival, and length of stay. There was significant heterogeneity in the reporting of mortality, with 13 individual outcome assessments used across studies. In-hospital mortality was reported most commonly followed by ICU mortality. Outcome assessments tended to focus on acute or early mortality, with few studies reporting mortality beyond 30 days. ICU and in-hospital length of stay were frequently reported across studies.

### Clinician-reported outcomes

Clinician-reported outcome (ClinRO) measures were the most commonly reported outcome measures overall. In total, ClinRO measures were reported a total of 101 times. The most frequently reported categories include heart rate control and or decrease, cardioversion or rhythm control (chemical or electrical direct-current cardioversion DCCV), arrhythmia recurrence, and outcome measures reporting on stroke and anticoagulation. Supplementary material online, *Table S3* (see Supplementary material) shows all ClinRO measures reported as primary or secondary outcomes stratified by individual study.

Cardioversion or rhythm control was the most commonly reported ClinRO measure. Cardioversion to sinus rhythm, followed by successful DCCV, where the most frequently reported measures, although the authors did not define successful DCCV. Whilst cardioversion was frequently reported, there was disagreement in the timing and cut-offs used for cardioversion to sinus rhythm (*Table 2*). The longest assessment period for the maintenance of sinus rhythm was 48 h.

Despite ClinRO measures relating to heart rate control or decrease being frequently reported, there was significant heterogeneity between studies on which individual measure was appropriate. The only ClinRO measures to be reported by more than one study were heart rate reduction without a decrease in arterial pressure and heart rate control. Whilst heart rate control and time to heart rate control were frequently reported, there was disagreement in the heart rate thresholds that defined control. Six ClinRO defined HR control using static heart rate thresholds, e.g. HR <120, while other studies reported percentage decrease from baseline e.g. decrease in HR >20%.

ClinRO measures reporting on stroke and anticoagulation comprised 18 outcome measures, with incidence of stroke being the most frequently reported, followed by thromboembolic events. Despite this, there was scarce reporting on the assessment of stroke or bleeding risk.

### Patient-reported outcomes

Patient-reported outcome measures were poorly reported across all studies. Individual PRO measures were reported four times, with all measures coming from one study. PRO measures assessed anxiety predominantly in the short-term using the hospital anxiety and depression scale (HADS), which typically assesses anxiety and depression symptoms in the previous week. Quality of life measures were assessed using the Short Form-36 (SF-36), but the authors did not report when they assessed the SF-36. The longest assessment of PRO measures was using the Impact of Event Scale-Revised (IES-R), which typically assesses the presence of post-traumatic stress disorder. The authors in this study followed up and reported PRO measures using the IES-R at 12 months. In addition, no studies reported economic outcomes such as healthcare utilization or cost, further limiting understanding of the broader impact of NOAF beyond the acute setting.

### Biomarker outcomes

Biomarker outcomes were reported relatively infrequently, with only three studies reporting Biomarker outcomes. Despite this, 25 individual biomarkers were reported as outcomes and included in *Table 3*.

### Performance-reported outcomes

Performance-reported outcomes rely on patients undertaking a task such as a functional 6-minute walk test, and reporting the response. No studies on the acute management of atrial fibrillation in critically unwell patients reported performance outcomes.

## Discussion

To the best of our knowledge, this is the first review to systematically map the current definitions used in clinical trials investigating NOAF in acutely unwell patients. We found marked heterogeneity in how NOAF was defined and diagnosed in ICU research, with variable arrhythmia duration thresholds, heart rate criteria, and monitoring methods. Outcome reporting was equally inconsistent. Clinician-reported outcomes (ClinRO) dominated, focusing mainly on rhythm control, heart rate control, and arrhythmia recurrence. Observer-reported outcomes (ObsRO), such as mortality and length of stay, were also commonly reported but varied in the timeframes and definitions used. Patient-reported outcomes (PRO), such as quality of life or mental health assessments, were notably underrepresented, reflecting a gap in understanding the patient-centric impacts of NOAF.

European guidelines define AF temporally as ‘First-diagnosed, Paroxysmal, Persistent or Permanent AF’.^1^ The term NOAF is used extensively in the critical care literature and typically denotes AF in a patient without a prior history of the arrhythmia. Despite its significance, there remain knowledge gaps on how to define, diagnose, and manage this common condition.

Our findings highlight heterogeneity between studies, such as the arrhythmias that are included in clinical trials, ECG criteria used to define AF, lack of standardized criteria of arrhythmia duration prior to diagnosis, and the variation in heart rate thresholds before initiation of clinical intervention. Some studies adhered to the European Society of Cardiology (ESC) guidelines requiring sustained atrial fibrillation for at least 30 s, while others adopted thresholds ranging from 15 min to one hour. Similarly, heart rate criteria prior to intervention ranged widely, with thresholds set between 100 bpm and 120 bpm, or omitted entirely. Despite the increasing number of publications in this field over time, we did not observe a clear trend towards greater consistency in the definitions of NOAF used in ICU-based research. Few studies defined inclusion criteria using definitions proposed by national and international societies.^1^ Studies often included more than one type of arrhythmia, rarely defined ECG characteristics required for the diagnosis of included arrhythmias, and reported a range of arrhythmia durations or heart rate limits prior to diagnosis or intervention. Addressing this variability in the definition of clinically important AF was recently highlighted as a research priority at an international symposium, with the recommendation that future studies should include a common definition that is flexible, pragmatic, and operational in the clinical setting.^16^ A key conceptual distinction emerging from this review is the differing role of NOAF definitions depending on whether NOAF is treated as an outcome or as an inclusion criterion. When NOAF is used as an outcome measure, standardization is essential to ensure comparability across studies and to enable meaningful synthesis of incidence and treatment effects. In contrast, when NOAF is used as an inclusion criterion, heterogeneity in definitions may be both expected and appropriate. For example, short-duration episodes of AF may be clinically relevant for studies investigating rate or rhythm control, whereas longer-duration or recurrent AF episodes may be required when studying outcomes such as stroke or long-term mortality. This distinction highlights that future consensus definitions should be flexible and context-specific rather than universally fixed.

Mapping the diagnostic criteria for NOAF is clinically important because inconsistent definitions directly affect which patients are identified, how quickly they are treated, and how trial results are interpreted. In ICU practice, variation in arrhythmia duration thresholds, heart rate cut-offs, and monitoring modalities can lead to significant differences in when NOAF is recognized and whether it is acted upon. This has direct implications for haemodynamic stability, anticoagulation decisions, and rhythm or rate control strategies. By systematically collating and comparing these diagnostic approaches, our review provides ICU clinicians with a clear picture of current practice variation, enabling them to benchmark their own diagnostic thresholds against those used internationally. It also highlights the need for adopting consistent, evidence-based definitions to improve patient selection for interventions, reduce unwarranted variation in care, and enhance the comparability of clinical studies. Current definitions of NOAF largely rely on rhythm characteristics such as duration and heart rate thresholds but fail to account for the underlying physiological context in which AF occurs. In critically ill patients, transient AF during procedural stimulation represents a fundamentally different clinical entity from AF occurring during septic shock or multi-organ failure. The absence of illness severity markers within definitions, therefore, contributes to heterogeneity in study populations and limits the applicability of findings. Future work should consider integrating measures of illness severity, haemodynamic instability, and underlying disease processes into definitions of clinically relevant NOAF.

Furthermore, the increasing use of large clinical databases and machine learning approaches to predict AF and related outcomes amplifies the importance of consistent definitions. Variable definitions of NOAF will significantly influence model training and performance, leading to inconsistent or non-reproducible prediction tools. Standardized definitions could improve model robustness and facilitate external validation. Additionally, future studies would benefit from incorporating sensitivity analyses using alternative AF thresholds to better understand the impact of definitional variability.

The heterogeneity identified in this review has important methodological implications. Variation in NOAF definitions may introduce misclassification bias, whereby patients with differing arrhythmia burden or clinical significance are grouped together. This may dilute observed treatment effects and reduce the internal validity of studies. Furthermore, inconsistent definitions limit external validity and the ability to generalize findings across settings. However, it is important to recognize where heterogeneity reflects appropriate adaptation to specific research questions. Distinguishing between necessary clinical flexibility and avoidable methodological inconsistency will be critical in the development of future consensus definitions and core outcome sets.

We also identified the limited use of biomarker outcomes, even though biomarkers could offer objective insights into the pathophysiological mechanisms and therapeutic impacts of NOAF. Furthermore, performance-reported outcomes were entirely absent, likely due to the acute nature of critical care and the challenges of assessing performance in this setting.

The lack of reporting of PROs underscores the importance of involving patients, families, and the public in defining outcomes that matter. Many critically ill patients will be unaware of having suffered from NOAF during their ICU stay, while clinicians may underestimate its long-term consequences, assuming it to be a benign marker of acute stress. Despite increasing recognition of the importance of patient-centred care, there remains a clear absence of patient and public involvement (PPI) in shaping outcome selection in this field. Incorporating patient perspectives is essential to ensure that future studies capture outcomes that reflect lived experience, including quality of life, functional recovery, and longer-term symptom burden. In addition, no studies reported economic outcomes, such as healthcare utilization or cost-effectiveness, which are critical for informing resource allocation and policy decisions. Future research, particularly in the development of a Core Outcome Sets, should integrate both PPI and economic evaluation to ensure that outcomes are clinically meaningful, patient-relevant, and aligned with healthcare system priorities.

There was a notable absence of long-term follow-up data related to patient-reported outcomes (PROs) in studies. Without information on quality of life, functional recovery, mental health, and longer-term cardiovascular events, our understanding of recovery trajectories remains incomplete. This limitation restricts our ability to identify which patients may benefit from long-term monitoring, anticoagulation, or rhythm-control strategies after ICU discharge. Given emerging evidence that NOAF during critical illness is associated with recurrent AF and increased risk of stroke, it is crucial to determine who should receive targeted follow-up and secondary prevention. The *Atrial fibrillation Better Care* (ABC) pathway, endorsed in international guidelines, emphasizes a holistic, integrated ABC approach (**A**: avoid stroke and anticoagulation, **B:** better symptom management, **C**: cardiovascular risk factor and comorbidity optimization).^70^ Applying the ABC pathway to post-ICU NOAF patients requires robust evidence on long-term outcomes and patient experience; however, this evidence is currently lacking due to the heterogeneity and short follow-up of existing studies.^71^ Addressing these gaps is essential for both personalizing long-term care and improving overall cardiovascular outcomes in this high-risk population.

The COS-ABACUS initiative (Core Outcome Set for Atrial Fibrillation in the Critically Unwell) directly responds to this gap.^72^ The results of this review will inform a Delphi consensus process involving key stakeholders, including patients, caregivers, ICU clinicians, cardiologists, and researchers.^72^ While the results of this review will inform future COS, the findings have direct applicability to current clinical practice. By providing a clear synthesis of how NOAF is presently defined and diagnosed in ICU-based research, clinicians can immediately benchmark their own diagnostic thresholds and monitoring strategies against those used internationally. This will help standardize recognition of clinically important NOAF at the bedside, improve consistency in treatment decisions, and guide post-discharge referral pathways, even before a formal consensus definition or COS is finalized.

## Conclusion

This scoping review underscores the urgent need for standardization in the definition and outcome reporting of NOAF in critically ill patients. Establishing a Core Outcome Set (COS) specific to this population is crucial to reducing heterogeneity and improving the comparability of study findings. Future research should prioritize consensus-building efforts to define NOAF and identify universally relevant outcomes.

Moreover, prospective studies and randomized controlled trials should adopt standardized methodologies, including the consistent use of validated biomarkers and PROs. These efforts will enhance the evidence base, facilitate meta-analyses, and ultimately guide more effective, evidence-based management strategies for NOAF in critically ill patients. Addressing these gaps will be critical to advancing clinical care and improving patient outcomes in this high-risk population.

## Supplementary Material

oeag075_Supplementary_Data

## Data Availability

Data available on request: *The data underlying this article will be shared on reasonable request to the corresponding author.*
