# Supplementary material for: Definitions and outcomes reported in studies investigating the management of new onset atrial fibrillation in acutely unwell patients admitted to the intensive care unit: a scoping review
Source: Eur Heart J Open. 2026 May 9;6(3):oeag075. doi: 10.1093/ehjopen/oeag075 (PMC13252546; doi:10.1093/ehjopen/oeag075)
Supplement: oeag075_Supplementary_Data [file oeag075_supplementary_data.zip › Supplementary Material_R .docx]

**Definitions and outcomes reported in studies investigating the management of New Onset Atrial Fibrillation in acutely unwell patients admitted to the Intensive Care Unit: A Scoping Review**

**SUPPLEMENTARY MATERIAL**

Brian W Johnston*^1,2,3^, Jobby Matthews^6^, Callum Voller^6^, Ruaraidh A Hill^4^, Bronagh Blackwood^9^, Karen Williams^3^, Angela Hall^3^, Keith Wilson^5^, Trudie H Lobban^10^, Gregory Y.H. Lip^1,2,7,8^ and Ingeborg D. Welters^1,2,3^.

**Affiliations:**

1. Department of Cardiovascular and Metabolic Medicine, Institute of Life Course and Medical Sciences, University of Liverpool, Liverpool, UK
2. Liverpool Centre for Cardiovascular Science at University of Liverpool, Liverpool John Moores University, Liverpool UK
3. University Hospitals of Liverpool Group, Royal Liverpool University Hospital, Department of Critical Care Medicine, Liverpool, UK
4. Liverpool Reviews and Implementation Group (LRiG), University of Liverpool, Department of Health Data Science, Institute of Population Health, Liverpool, UK
5. Liverpool Heart and Chest Hospital NHS Foundation Trust, Liverpool, UK
6. Liverpool School of Medicine, University of Liverpool, Liverpool, UK
7. Department of Clinical Medicine, Aalborg University, Aalborg, Denmark
8. Medical University of Bialystok, Bialystok, Poland
9. Wellcome Wolfson Institute for Experimental Medicine, Queens University Belfast, Belfast, UK
10. Arrhythmia alliance, Heart Rhythm Alliances and STARS, UK

**Supplementary Material Figure 1. PRISMA statement of Included studies**

Studies from databases/registers **(n = 8477)**

Embase (n = 6782)

MEDLINE (n = 1665)

Johnston et al 2022 (n = 30)

Studies excluded **(n = 6230)**

Studies not retrieved **(n = 0)**

Studies assessed for eligibility **(n = 275)**

Studies sought for retrieval **(n = 275)**

Studies screened **(n = 6505)**

Studies excluded **(n = 182)**

Wrong setting (n = 2)

Wrong outcomes (n = 36)

Duplicate study (n = 11)

Wrong intervention (n = 28)

Wrong study design (n = 100)

Wrong patient population (n = 5)

**Identification**

References removed **(n = 1972)**

Duplicates identified manually (n = 40)

Duplicates identified by Covidence (n = 1932)

Marked as ineligible by automation tools (n = 0)

**Screening**

**Included**

**Included**

Studies included in full text review **(n = 93)**

Studies excluded **(n = 49)**

Wrong outcomes (n = 11)

Wrong intervention (n =12)

Wrong study design (n = 18)

Wrong patient population (n = 4)

Wrong setting (n=4)

Studies included in review **(n=44)**

**Supplementary Material Table 1. Full search strategy**

**Emcare search strategy:**

| # | Query | Results from 16 Jun 2025 |
| --- | --- | --- |
| 1 | Atrial Fibrillation/ | 38,034 |
| 2 | (Atrial* adj2 (Fibrillat* or flutter*)).tw. | 43,897 |
| 3 | AF.tw. | 22,741 |
| 4 | (tachycardia or tachyarrhythmia or arrhythmia or supraventricular).tw. | 39,567 |
| 5 | 1 or 2 or 3 or 4 | 97,028 |
| 6 | ((new* or recent*) adj1 diagno*).tw. | 34,158 |
| 7 | (onset* or new*).tw. | 1,168,952 |
| 8 | 6 or 7 | 1,171,469 |
| 9 | Critical Care/ | 39,648 |
| 10 | Critical Illness/ | 17,065 |
| 11 | ((critical* or intensiv*) adj4 (care or ill*)).tw. | 170,298 |
| 12 | ((critical* or intensiv*) adj4 (care or ill* or unwell*)).tw. | 170,383 |
| 13 | 9 or 10 or 11 or 12 | 187,166 |
| 14 | Emergency Medicine/ | 20,763 |
| 15 | (Accident adj2 Emergency).tw. | 3,048 |
| 16 | "Emergency Department* ".tw. | 90,731 |
| 17 | (casualty adj2 department).tw. | 134 |
| 18 | "Emergency Room* ".tw. | 11,800 |
| 19 | 14 or 15 or 16 or 17 or 18 | 118,022 |
| 20 | "Acute Medical Unit* ".tw. | 303 |
| 21 | AMU.tw. | 318 |
| 22 | "Acute Medical Assessment Unit* ".tw. | 17 |
| 23 | "Acute Medicine".tw. | 523 |
| 24 | AMAU.tw. | 8 |
| 25 | "Acute Medical Admission* Unit* ".tw. | 18 |
| 26 | 20 or 21 or 22 or 23 or 24 or 25 | 1,075 |
| 27 | Perioperative Care/ | 2,023 |
| 28 | "Perioperative patient* ".tw. | 730 |
| 29 | "perioperative population* ".tw. | 32 |
| 30 | 27 or 28 or 29 | 2,744 |
| 31 | (surg* adj2 patient*).tw. | 75,225 |
| 32 | "surgical patient* ".tw. | 12,828 |
| 33 | "surgical population* ".tw. | 963 |
| 34 | 31 or 32 or 33 | 75,827 |
| 35 | 13 or 19 or 26 or 30 or 34 | 367,775 |
| 36 | 5 and 8 and 35 | 1,408 |
| 37 | 5 and 8 | 15,972 |
| 38 | 13 and 37 | 661 |
| 39 | 19 or 26 | 118,946 |
| 40 | 37 and 39 | 564 |
| 41 | 30 or 34 | 78,101 |
| 42 | 37 and 41 | 290 |

**Embase Search Strategy:**

| # | Query | Results from 16 Jun 2025 |
| --- | --- | --- |
| 1 | Atrial Fibrillation/ | 235,646 |
| 2 | (Atrial* adj2 (Fibrillat* or flutter*)).tw. | 196,820 |
| 3 | AF.tw. | 118,663 |
| 4 | (tachycardia or tachyarrhythmia or arrhythmia or supraventricular).tw. | 193,722 |
| 5 | 1 or 2 or 3 or 4 | 453,177 |
| 6 | ((new* or recent*) adj1 diagno*).tw. | 196,331 |
| 7 | (onset* or new*).tw. | 5,871,550 |
| 8 | 6 or 7 | 5,882,974 |
| 9 | Critical Care/ | 163,339 |
| 10 | Critical Illness/ | 40,789 |
| 11 | ((critical* or intensiv*) adj4 (care or ill*)).tw. | 464,762 |
| 12 | ((critical* or intensiv*) adj4 (care or ill* or unwell*)).tw. | 465,048 |
| 13 | 9 or 10 or 11 or 12 | 532,300 |
| 14 | Emergency Medicine/ | 49,931 |
| 15 | (Accident adj2 Emergency).tw. | 6,973 |
| 16 | "Emergency Department* ".tw. | 229,746 |
| 17 | (casualty adj2 department).tw. | 735 |
| 18 | "Emergency Room* ".tw. | 50,981 |
| 19 | 14 or 15 or 16 or 17 or 18 | 316,401 |
| 20 | "Acute Medical Unit* ".tw. | 903 |
| 21 | AMU.tw. | 2,056 |
| 22 | "Acute Medical Assessment Unit* ".tw. | 116 |
| 23 | "Acute Medicine".tw. | 1,429 |
| 24 | AMAU.tw. | 101 |
| 25 | "Acute Medical Admission* Unit* ".tw. | 93 |
| 26 | 20 or 21 or 22 or 23 or 24 or 25 | 4,211 |
| 27 | Perioperative Care/ | 5,904 |
| 28 | "Perioperative patient* ".tw. | 1,911 |
| 29 | "perioperative population* ".tw. | 54 |
| 30 | 27 or 28 or 29 | 7,772 |
| 31 | (surg* adj2 patient*).tw. | 330,268 |
| 32 | "surgical patient* ".tw. | 43,335 |
| 33 | "surgical population* ".tw. | 3,076 |
| 34 | 31 or 32 or 33 | 332,094 |
| 35 | 13 or 19 or 26 or 30 or 34 | 1,135,678 |
| 36 | 5 and 8 and 35 | 7,810 |
| 37 | 5 and 8 | 83,596 |
| 38 | 13 and 37 | 3,437 |
| 39 | 19 or 26 | 320,083 |
| 40 | 37 and 39 | 3,397 |
| 41 | 30 or 34 | 338,439 |
| 42 | 37 and 41 | 1,657 |

**MEDLINE/PubMed search strategy:**

| # | Query | Results from 16 Jun 2025 |
| --- | --- | --- |
| 1 | Atrial Fibrillation/ | 78,330 |
| 2 | (Atrial* adj2 (Fibrillat* or flutter*)).tw. | 106,719 |
| 3 | AF.tw. | 61,165 |
| 4 | (tachycardia or tachyarrhythmia or arrhythmia or supraventricular).tw. | 119,459 |
| 5 | 1 or 2 or 3 or 4 | 238,629 |
| 6 | ((new* or recent*) adj1 diagno*).tw. | 104,823 |
| 7 | (onset* or new*).tw. | 4,560,121 |
| 8 | 6 or 7 | 4,566,571 |
| 9 | Critical Care/ | 63,795 |
| 10 | Critical Illness/ | 43,226 |
| 11 | ((critical* or intensiv*) adj4 (care or ill*)).tw. | 303,361 |
| 12 | ((critical* or intensiv*) adj4 (care or ill* or unwell*)).tw. | 303,494 |
| 13 | 9 or 10 or 11 or 12 | 330,217 |
| 14 | Emergency Medicine/ | 15,923 |
| 15 | (Accident adj2 Emergency).tw. | 5,193 |
| 16 | "Emergency Department* ".tw. | 145,560 |
| 17 | (casualty adj2 department).tw. | 579 |
| 18 | "Emergency Room* ".tw. | 26,706 |
| 19 | 14 or 15 or 16 or 17 or 18 | 184,646 |
| 20 | "Acute Medical Unit* ".tw. | 468 |
| 21 | AMU.tw. | 1,611 |
| 22 | "Acute Medical Assessment Unit* ".tw. | 37 |
| 23 | "Acute Medicine".tw. | 817 |
| 24 | AMAU.tw. | 21 |
| 25 | "Acute Medical Admission* Unit* ".tw. | 46 |
| 26 | 20 or 21 or 22 or 23 or 24 or 25 | 2,799 |
| 27 | Perioperative Care/ | 17,412 |
| 28 | "Perioperative patient* ".tw. | 1,282 |
| 29 | "perioperative population* ".tw. | 32 |
| 30 | 27 or 28 or 29 | 18,474 |
| 31 | (surg* adj2 patient*).tw. | 208,358 |
| 32 | "surgical patient* ".tw. | 29,780 |
| 33 | "surgical population* ".tw. | 1,838 |
| 34 | 31 or 32 or 33 | 209,527 |
| 35 | 13 or 19 or 26 or 30 or 34 | 715,246 |
| 36 | 5 and 8 and 35 | 2,779 |
| 37 | 5 and 8 | 39,028 |
| 38 | 13 and 37 | 1,208 |
| 39 | 19 or 26 | 187,197 |
| 40 | 37 and 39 | 985 |
| 41 | 30 or 34 | 225,317 |
| 42 | 37 and 41 | 759 |

| **Supplementary Material Table 2. ISPOR Framework Categories** | | |
| --- | --- | --- |
| **ISPOR Terminology** | **Definition / alternative terminology** | **Examples related to NOAF** |
| **Clinical outcome assessment:** Clinical outcome assessments rely upon the patient or another person to integrate observations and report the results. | | |
| **Observed reported outcome (ObsRO)** | Assessment where observations can be recorded by a person other than the patient and no experience is required to quantify the outcome | 1) Intensive care length of stay  2) Hospital length of stay  3) Mortality |
| **Performance outcome (PerfO)** | Patient performs a task but no rater or perspective or clinical judgement is needed to quantify the performance | 1) Functional 6-minute walk test |
| **Patient reported outcome (PRO)** | Assessments that rely directly on patients’ response (without further interpretation from a clinician) | 1) Hospital Anxiety and Depression scale |
| **Clinician reported outcome (ClinRO)** | Relies on the appropriate health care professional to be the rater. Clinician required to provide professional expertise to the observation or is needed to interpret the patient’s response, actions or state | 1) CHA_2_DS_2_VASc score  2) HASBLED score  3) ORBIT score |
| **Biomarker outcome:** Biomarker outcomes are clinical laboratory measures that may be used in clinical practice or clinical trial protocols | | |
| **Biomarker outcome** | A biochemical measure that is not subject to patient or researchers/rater interpretation | 1) HbA1c  2) Troponin  3) Creatinine |

| Supplementary Material Table 3. Primary and secondary outcomes and associated ISPOR category of included studies | | | |
| --- | --- | --- | --- |
|  | **Author**  **Year** | **Primary outcomes** | **Secondary Outcomes** |
| **Randomised controlled trials** | | | |
| **1** | **Kakihana et al**  **(2020)** | HR control (defined as HR 60-94bpm after 24 hours) **(ClinRO)** | Decrease in HR >20% or more at 24hr, 48hr, 72hr and 96hr **(ClinRO)**  ICU free days at 28d **(ObsRO)**  Hospital free days at 28d **(ObsRO)**  28d mortality **(ObsRO)**  Lactate, creatinine kinase, troponin-I, BNP, arterial pH, BE, PF ratio, PaCO2, eGFR **(Biomarker)** |
| **2** | **Delle Karth et al**  **(2001)** | Cardioversion to SR **(ClinRO)** | HR reduction >30% of baseline for within a 4hr period and sustained for >4hr **(ClinRO)**  Controlled tachycardia (HR <120) **(ClinRO)**  Uncontrolled tachycardia (HR >120 within 4 hr of study drug administration) **(ClinRO)** |
| **3** | **Balser et al**  **(1998)** | Cardioversion to SR at 2hr and 12hr **(ClinRO)** | HR (reported but no definition of successful HR control) **(ClinRO)**  ICU LoS (**ObsRO**)  In-hospital mortality (**ObsRO** |
| **4** | **Moran et al**  **(1995)** | Cardioversion to SR within 24hr **(ClinRO)**  HR (reported but no definition of successful HR control) **(ClinRO)** | n/r |
| **Prospective Observational Studies** | | | |
| **5** | **Uchino et al**  **(2020)** | In-hospital mortality in NOAF lasting >48 hours vs NOAF lasting <48 hours **(ObsRO)** | Hospital LoS **(ObsRO)**  Incidence of stroke **(ClinRO)** |
| **6** | **Meierhenrich et al**  **(2010)** | Cardioversion to SR **(ClinRO)** | Arrhythmia recurrence rate **(ClinRO)**  ICU mortality **(ObsRO)**  28d mortality **(ObsRO)**  60d mortality **(ObsRO)**  CRP, WCC **(Biomarker**) |
| **7** | **Gerlach et al**  **(2008)** | Cardioversion to SR at 24 hours **(ClinRO)** | Time to cardioversion **(ClinRO)** |
| **8** | **Milicevic et al**  **(2008)** | Cardioversion to SR following sequential administration of up to 3 antiarrhythmics **(ClinRO)** | Time to cardioversion **(ClinRO)** |
| **9** | **Sleeswijk et al**  **(2008)** | Cardioversion to SR **(ClinRO)** | HR control (<110 bpm) **(ClinRO)**  Time to cardioversion **(ClinRO)**  Time to HR control **(ClinRO)** |
| **10** | **Hayashi**  **(2005)** | Cardioversion to SR following DCCV failure (failure to maintain cardioversion following 3xDCCV shocks) **(ClinRO)** | n/r |
| **11** | **Mayr**  **(2003)** | Cardioversion to SR **(ClinRO)** | n/r |
| **12** | **Hennersdorf**  **(2002)** | Termination of arrhythmia in <90 min of Ibutilide infusion **(ClinRO)** | n/r |
| **13** | **Song**  **(2023)** | Occurrence of NOAF with 7d of ICU admission **(ClinRO)** | Time from ICU admission to NOAF **(ClinRO)**  ICU LoS **(ObsRO)**  Hospital LoS **(ObsRO)**  In-hospital mortality **(ObsRO)**  Adverse events associated with Dexmedetomidine **(ClinRO)**  Treatment of AF **(ClinRO)** |
| **14** | **Makrygiannis**  **(2018)** | All-cause mortality during ICU **(ObsRO)** | ICU LoS **(ObsRO)** |
| **15** | **Arrigo**  **(2018)** | All-cause 1 year mortality **(ObsRO)** | Incidence of cardiovascular morbidity **(ClinRO)**  Incidence of cardiovascular mortality **(ClinRO)**  Quality of life 1 year post ICU*** **(PRO)**  Biomarkers in long term outcome post  ICU**** **(Biomarker outcome)** |
| **16** | **Wetterslev (2023)** | Occurrence of AF and NOAF **(ClinRO)** | Hospital LoS **(ObsRO)**  90d mortality **(ObsRO)**  Ischaemic or thromboembolic events **(ClinRO)**  Severe bleeding events **(ClinRO)** |
| **Retrospective Observational Studies** | | | |
| **17** | **Rottmann**  **(2024)** | ICU survival **(ObsRO)** | Hospital survival **(ObsRO)**  Length of stay – not defined ICU or hospital **(ObsRO)**  Readmission to ICU **(ObsRO)**  Hours of mechanical ventilation **(ClinRO)**  Use of ECMO **(ClinRO)**  Needs for dialysis **(ClinRO)** |
| **18** | **Bedford**  **(2022)** | Time to rate control (HR <110bpm) **(ClinRO)**  Time to rhythm control **(ClinRO)** | 30d mortality **(ObsRO)** |
| **19** | **Gillmann**  **(2022)** | Time to reach HR <110 bpm for 1 hour **(ClinRO)** | Heart rate delta relative to medication start **(ClinRO)**  Hours in sinus rhythm within first 24 hours **(ClinRO)**  Hours until sinus rhythm conversion **(ClinRO)**  Vital parameters at sinus rhythm conversion **(ClinRO)**  Changes in haemodynamic stability **(ClinRO)**  Occurrence of bradycardic episodes (ClinRO |
| **20** | **Miller**  **(2022)** | Incidence of therapeutic anticoagulation **(ClinRO)** | Thromboembolic events **(ClinRO)**  Bleeding events **(ClinRO)**  ICU LoS **(ObsRO)**  Hospital LoS **(ObsRO)**  Basic/advanced respiratory support duration **(ClinRO)**  ICU mortality **(ObsRO)**  In-hospital mortality **(ObsRO)**  HASBLED score **(ClinRO)**  CHA_2_DS_2_VASc score **(ClinRO)** |
| **21** | **Suresh**  **(2022)** | Conversion to SR **(ClinRO)** | AF recurrence in 24 hours **(ClinRO)**  AF recurrence after 24 hours of SR **(ClinRO)**  Adverse events:  Cerebrovascular accident **(ClinRO)**  Encephalopathy **(ClinRO)**  Myocardial infarction **(ClinRO)**  30d mortality **(ObsRO)**  Drug related adverse events **(ClinRO)** |
| **22** | **Shima**  **(2021)** | Success rate of DCCV **(ClinRO)** | ICU mortality **(ObsRO)**  In-hospital mortality **(ObsRO)**  Cardiac rhythm at ICU discharge **(ClinRO)**  ICU LoS **(ObsRO)**  Hospital LoS **(ObsRO)**  Incidence stroke **(ClinRO)**  Bleeding events **(ClinRO)** |
| **23** | **Yoshida**  **(2021)** | Last SR restoration (within 7 days after the initial AF onset) **(ClinRO)** | Cardiac rhythm at ICU discharge **(ClinRO)**  AF duration **(ClinRO)**  ICU LoS **(ObsRO)**  Hospital LoS **(ObsRO)**  ICU mortality **(ObsRO)**  In-hospital mortality **(ObsRO)**  Incidence of stroke **(ClinRO)** |
| **24** | **Jacobs**  **(2020)** | In-hospital mortality **(ObsRO)** | 1-year mortality **(ObsRO)**  Morality at maximum follow up **(ObsRO)**  ICU LoS **(ObsRO)**  Hospital LoS **(ObsRO)** |
| **25** | **Betthauser**  **(2019)** | Descriptive use of amiodarone **(ClinRO)** | ICU LoS **(ObsRO)** |
| **26** | **Herasevich**  **(2019)** | Impact of digoxin on haemodynamic profile (including HR pre and post digoxin at 6, 12, 24 hrs) **(ClinRO)** | In-hospital mortality **(ObsRO)**  Hospital LoS **(ObsRO)**  ICU mortality **(ObsRO)**  ICU LoS **(ObsRO)** |
| **27** | **Kim**  **(2019)** | First occurrence of ischaemic stroke/systemic emboli **(ClinRO)** | All-cause mortality **(ObsRO)**  6-month mortality **(ObsRO)** |
| **28** | **Kyo et al**  **(2019)** | Success rate of DCCV **(ClinRO)**  ICU mortality **(ObsRO)** | ICU LoS **(ObsRO)**  ICU mortality **(ObsRO)**  Hospital LoS **(ObsRO)**  In-hospital mortality **(ObsRO)**  Incidence of stroke **(ClinRO)**  DCCV energy **(ClinRO)**  Number of shocks during DCCV **(ClinRO)**  Time from AF to DCCV **(ClinRO)**  Success rate of DCCV **(ClinRO)**  AF recurrence after DCCV **(ClinRO)**  Incidence of stroke **(ClinRO)**  Hospital LoS **(ObsRO)**  ICU LoS **(ObsRO)** |
| **29** | **Milojevic et al**  **(2019)** | Cardioversion to SR within 40 min of treatment initiation **(ClinRO)** | HR control <100bpm **(ClinRO)** |
| **30** | **Schoaps**  **(2019)** | Prescription of oral anticoagulant on hospital discharge (in NOAF with CHA_2_DS_2_VASc >2) **(ClinRO)** | ICU LoS **(ObsRO)**  Hospital LoS **(ObsRO)**  Reason for non-prescription OAC **(ClinRO)**  Association between individuals CHA_2_DS_2_VASc components and OAC prescription **(ClinRO)** |
| **31** | **Brown et al**  **(2018)** | Cardioversion to SR within 48 **(ClinRO)** | HR control (reported but no definition of successful HR control) hours **(ClinRO)**  ICU LoS **(ObsRO)**  Hospital LoS **(ObsRO)** |
| **32** | **Yoshida et al**  **(2018)** | Cardioversion to SR within 6 hours **(ClinRO)** | In-hospital mortality **(ObsRO)**  Incidence of stroke **(ClinRO)** |
| **33** | **Balik et al**  **(2017)** | Cardioversion to SR within 24 hours **(ClinRO)** | ICU mortality **(ObsRO)**  28d mortality **(ObsRO)**  1-year mortality **(ObsRO)** |
| **4** | **Duby et al**  **(2017)** | Mortality **(ObsRO)** | ICU LoS **(ObsRO)**  Hospital LoS **(ObsRO)**  Thromboembolic events **(ClinRO)** |
| **35** | **Liu et al**  **(2016)** | Cardioversion to SR **(ClinRO)** | In-hospital mortality **(ObsRO)** |
| **36** | **Mitric et al**  **(2016)** | Cardioversion to SR within 12 hrs **(ClinRO)** | n/r |
| **37** | **Walkey et al**  **(2016)** | In-hospital mortality **(ObsRO)** | n/r |
| **38** | **Okajima et al**  **(2015)** | Cardioversion to SR **(ClinRO)** | HR reduction without decrease in arterial pressure (successful HR reduction not defined) **(ClinRO)** |
| **39** | **Xie et al**  **(2015)** | Cardioversion to SR **(ClinRO)** | 30d mortality **(ObsRO)** |
| **40** | **Personett et al**  **(2014)** | HR control (defined as HR <110 beats/min and SBO >90mmHg for 6hrs **(ClinRO)** | ICU LoS **(ObsRO)**  Readmission to ICU for AF **(ClinRO)** |
| **41** | **Kanji et al**  **(2012)** | Rhythm control **(ClinRO)**  HR control **(ClinRO)** | In-hospital mortality **(ObsRO)**  ICU mortality **(ObsRO)**  Hospital LoS **(ObsRO)**  ICU LoS **(ObsRO)**  Thromboembolic events **(ClinRO)** |
| **42** | **Delle Karth**  **(2005)** | Termination of arrhythmia within 60 min **(ClinRO)** | n/r |
| **43** | **Mayr et al**  **(2004)** | Cardioversion to SR **(ClinRO)**  Heart rate at 12, 24 and 48 hours **(ClinRO)** | n/r |
| **44** | **Varriale et al**  **(2000)** | Cardioversion to SR **(ClinRO)** | n/r |
